# Evaluating the effect of the Practical Approach to Care Kit on teaching medical students primary care: Quasi-experimental study

**DOI:** 10.4102/phcfm.v9i1.1602

**Published:** 2017-12-08

**Authors:** Robert Mash, Michael Pather, Hilary Rhode, Lara Fairall

**Affiliations:** 1 Division of Family Medicine and Primary Care, Stellenbosch University, South Africa; 2 Knowledge Translation Unit, Lung Institute, University of Cape Town, South Africa

## Abstract

**Background:**

South Africa is committed to health reforms that strengthen primary health care. Preparing future doctors to work in primary care teams with other professionals is a priority, and medical schools have shifted towards community-based and decentralised training of medical students.

**Aim:**

To evaluate the effect on student performance of the Practical Approach to Care Kit (PACK) (an integrated decision-making tool for adult primary care) during the final phase of medical student training at Stellenbosch University.

**Setting:**

Clinical rotations in family medicine at clinics in the Western Cape.

**Methods:**

Mixed methods involving a quasi-experimental study and focus group interviews. Student examination performance was compared between groups with and without exposure to the PACK during their clinical training. Student groups exposed to PACK were interviewed at the end of their rotations.

**Results:**

Student performance in examinations was significantly better in those exposed to the PACK. Students varied from using the PACK overtly or covertly during the consultation to checking up on decisions made after the consultation. Some felt that the PACK was more suitable for nurses or more junior students. Although tutors openly endorsed PACK, very few modelled the use of PACK in their clinical practice.

**Conclusion:**

The use of PACK in the final phase of undergraduate medical education improved their performance in primary care. Students might be more accepting and find the tool more useful in the earlier clinical rotations. Supervisors should be trained further in how to incorporate the use of the PACK in their practice and educational conversations.

## Introduction

The health system in South Africa is in the process of transformation with an emphasis on re-engineering primary health care to achieve universal coverage and a more equitable system funded through national health insurance.^1,2^ The Ideal Clinic initiative is committed to patients having access to a competent primary care doctor as part of a multidisciplinary team.^3^ In the community, this team should consist of community health workers coordinated by a nurse and responsible for a specific number of households in a defined geographical area.^1^ These teams would be supported by primary care facilities that are largely nurse-driven, but supported by doctors and family physicians. In South Africa, there is no requirement for doctors to train as generalists in order to work in primary care, although family medicine training was recognised in 2007 and the number of family physicians with four years of postgraduate training is slowly increasing.^4^

The educational system should be responsive to the needs of the population and the requirements of the health system.^5^ Over the last few decades, medical schools have reformed their undergraduate curricula to include attention to family medicine and primary care and a more disseminated community-based educational experience. At Stellenbosch University, there was no exposure to primary care in 1997, but by 2016 medical students had 13 weeks of exposure over the 6-year period.

Internationally, there has also been a move towards more community-based and socially responsive medical education. Two fundamental reforms have been proposed. Firstly, instructional reform to move from informative to formative and finally transformative education. The focus shifts from only developing knowledge and skills, to building professionals with congruent values, to creating professionals with the ability to be leaders and change agents. Secondly, institutional reform promotes a move towards sharing ideas and resources within a network of institutions in order to produce higher quality educational systems that still train people appropriately for the local context. In a resource-constrained health system with under-developed primary health care, all of these reforms make sense in the African context.

Within this context, the Knowledge Translation Unit at the University of Cape Town produced the Practical Approach to Care Kit (PACK) to support decision-making by primary care providers.^6^ The tool synthesises global evidence and local guidelines and policies into a simple-to-use integrated guide that deals comprehensively with 70 common symptoms and 8 groups of chronic conditions seen in adults in South African primary care. The South African health system has embraced the tool for use in primary care mainly through in-service training provided to nurse clinicians, and it has also been adapted for use in other countries such as Botswana, Malawi, Gambia, Brazil and Mexico.^6^ It makes sense therefore for the educational system to introduce medical students to the tool during their undergraduate studies in order to strengthen their competency in primary care and prepare them for future service as interns and junior doctors or for future postgraduate training as family physicians working alongside nurse clinicians.

The development of clinical competency during undergraduate education can be regarded as having four stages as shown in Table 1.^7^ Conceptually, the PACK should help medical students to move through the stages more effectively and to develop at least a level of conscious competence appropriate to newly qualified doctors.

**TABLE 1:** Four stages of competence model.

Stage of competence	Unconscious incompetence	Conscious incompetence	Conscious competence	Unconscious competence
Definition	The individual does not understand or know how to do something and does not necessarily recognise the deficit.	Although the individual does not understand or know how to do something, he or she does recognise the deficit, as well as the value of a new skill in addressing the deficit.	The individual understands or knows how to do something. However, demonstrating the skill or knowledge requires concentration.	The individual has had so much practice with a skill that it has become ‘second nature’ and can be performed easily.

*Source*: Adams^7^

Teaching in the clinical environment has many challenges such as balancing obligations to students and patients while working under pressures of time and workload.^8^ In this environment, the PACK could potentially assist the clinical trainer with some of the key aspects of clinical teaching such as the need to move beyond recall of knowledge to application in problem-solving, to enable self-directed learning and self-assessment as well as to probe for underlying evidence.

One of the key aspects of the PACK is the task-shifting of prescribing for high-burden non-communicable diseases like hypertension and diabetes from doctors to nurses, following similar initiatives to scale up infectious disease care.^9^ Such task-sharing requires increased collaboration between doctors and nurses, for which undergraduates require specific preparation.^5^ Existing exposure to nurse professionals in the undergraduate medical curriculum is dominated by experience with nurses in inpatient settings. There are limited opportunities for them to engage with nurse educators and very little opportunity to engage with the nurse clinicians who conduct most consultations in primary care settings.

The PACK is the first integrated decision support tool of its kind produced in the local context. Previously, the undergraduate curriculum at Stellenbosch University did not have any such integrated resource for primary care and directed the students to a variety of different resources. The impact of the PACK has been studied in the health system,^10,11,12^ but there has been no research looking at its impact in the educational system and no similar studies were identified in the literature.

This study aimed to evaluate the effect on student performance of implementing the PACK by means of an inter-professional training model during the student internship (final phase clinical rotation) in family medicine and primary care.

## Methods

### Study design

Mixed methods included a quasi-experimental study comparing student performance in those with and without exposure to the PACK as well as a phenomenological approach to qualitatively explore the students’ experience and perceptions of using PACK.

### Setting

The study was conducted in the sixth and final year of the MBChB programme at Stellenbosch University during 2014. The sixth year included nine 5-week clinical rotations in family medicine, primary care, community health and rehabilitation. Students in a rotation were allocated in groups of two to four students to a single site where they spent the whole five-weeks together. Possible public sector sites included eight community health centres and three district hospitals in the Western Cape and one district hospital in the Eastern Cape. At the facility, students worked under the supervision of a family physician alongside the primary care team. The rotation was structured so that students consulted ambulatory primary care patients during the morning. During the afternoon, they worked on quality improvement projects, performed home visits or might be on call in the emergency centre. Clinical rotations did not include any formal lectures or campus-based theoretical teaching.

At the end of each clinical rotation, students were assessed in a multiple choice examination, consisting of 30 single best answer questions, to test their application of knowledge to typical primary care scenarios.

At the end of each semester, students were assessed in an objective structured clinical examination (OSCE) with 11 stations of 5 min each that was designed to evaluate their consultation and clinical skills in primary care. The OSCE examination at the end of the first semester examined the students that did the rotation at the end of their fifth year and the students that did the rotation during the first three rotations of sixth year. The OSCE examination at the end of the second semester examined the sixth-year students from the fourth to the ninth rotations.

### Study population, sample size and sampling

All 190 student interns in their sixth year of study in 2014 were invited to participate in the study. There was no sampling and all agreed to participate. The PACK was introduced in rotations six to nine, and therefore these 83 students were the intervention group and the 64 students in rotations one to five were the control group. The 43 students that completed the rotation in their fifth year were unexposed to PACK and part of the first semester OSCE.

A power calculation was based on an end-of-block multiple choice questions (MCQ) test result from students who completed the test in early 2013. These students scored a mean of 69% [standard deviation (SD) 8%]. A sample size of 84 would be needed to detect a difference of half a SD with 90% power and type 1 error of 5%.

### Intervention

Students in the control group (rotations one to five) continued to consult patients under supervision without exposure to PACK, whereas students in the intervention group (rotations six to nine) were expected to use the PACK as explained below.

The PACK guide consisted of two main sections. The first section included approaches to 70 common reasons for encounter in primary care and the second section approaches to the management of eight groups of chronic conditions in primary care: HIV, TB, respiratory diseases, diseases of lifestyle, mental health, epilepsy, musculoskeletal and women’s health. The sections were cross-referenced with each other to support an integrated approach to care. The sections also differentiated which aspects of care were appropriate for a nurse clinician or a doctor. The guide represented a synthesis of best practice evidence that has also been adapted for use with the resources available in our specific primary care context.

In preparation for the introduction of PACK, the family physician supervisors were familiarised with the tool and how to use it with the medical students. This was done through a series of workshops during the last part of the first semester. Nurse clinician trainers were also employed and trained to provide interactive educational workshops at the training sites to medical students.

Each student in the intervention groups received a copy of the full guide printed in colour at the introduction to their rotation on campus. An orientation to the PACK was also given during the introduction. This orientation highlighted the rate of knowledge production in health sciences and the role of clinical decision support tools in providing evidence-informed care. It also showcased the evidence base underpinning the guide and the processes that were followed in synthesising evidence and tailoring recommendations for resource-constrained environments. The orientation was aimed at promoting the importance of an all-learning, as opposed to all-knowing, paradigm, reducing expectations that health professionals, in particular doctors, are expected ‘to know everything’, and promoted inter-professional collaboration with fellow nurse clinicians.

Once the students had arrived at their training sites, additional training was given by a nurse clinician trainer during weeks two, three and four of the rotation over the course of one morning per week. The aim of these interactive workshops was to further familiarise the students with using key sections of the guide, model inter-professional collaboration and create an opportunity to develop a first-name relationship with a nurse clinician. The nurse clinician trainers presented paper-based cases to the students and worked through the guide using these scenarios which were designed to highlight the need for better coordination between professionals and the place of standardised care in settings with limited continuity.

Student interns were expected to make use of the guide during their supervised consultations during the rotation at the training site. Family physicians were also expected to use the guide actively during their educational interactions and supervision of students over the whole five weeks.

### Data collection

The students’ scores in the end-of-rotation MCQ exam were used to assess their application of knowledge to clinical decision-making in typical primary care scenarios. Thirty single-answer MCQs were randomly selected at the end of each rotation from a bank of 60 MCQs for rotations one, two, three or four, and five in the control group in order to make four slightly different examinations. The same selected MCQs were then used in four corresponding rotations six to nine for the intervention group (i.e. rotations one and six sat the same examination and so on). Multiple choice questions were delivered to the class in the computer laboratory using Moodle software. The MCQ bank was blueprinted against the symptoms and chronic conditions included in the PACK and listed in the study guide for all students. Multiple choice questions content was aligned with the recommendations of PACK.

The OSCE at the end of the semester contained 11 stations that required the students to demonstrate their competency at assessing common presentations or managing common conditions in primary care. The same stations were used in both examinations to allow a more standardised comparison, although students would not have anticipated this. The aggregated scores from these selected stations were used to compare the performance of students in the intervention and control groups.

At the end of each of the four intervention rotations, a focus group interview was conducted with the students to explore their perceptions of the PACK and how it was used in the clinical context. The interview also explored their views on the nurse-led workshops. These interviews were conducted by H.R. using an interview guide. She was not one of the family physician tutors, but did conduct a few of the nurse-led workshops at the clinical sites.

### Data analysis

Data from the end-of-rotation MCQ exams were captured in an Excel spreadsheet and checked for errors. Students in the control group from rotations one to four were compared with students in the intervention group from rotations six to nine. Rotation five was excluded to avoid any contamination during the crossover period to use of the PACK.

Data from the OSCE were similarly captured in an Excel spreadsheet. Students in the control group from the first semester exam were compared to students in the second semester exam. Rotations four and five were excluded in the second semester exam as they had not been formally exposed to the PACK, but might have been contaminated by their peers when preparing for the exam.

Data were analysed using SPSS version 24. Descriptive statistics were reported on mean scores and SD for the MCQ and OSCE. The MCQ data were compared using an independent samples *t* test and the OSCE data with the Mann–Whitney *U* test as the data were not normally distributed.

Qualitative data were transcribed verbatim, checked against the audiotapes, and analysed with the help of Atlas-ti using the framework method.^13^ The framework method consisted of five steps: familiarisation with the data, creation of a thematic index, coding of data using the index, creation of charts and finally interpretation of the data to identify themes inductively.

The qualitative data analysis was triangulated with the quantitative data analysis to make sense of the effect on student performance of implementing the PACK.

### Ethical consideration

Ethics approval was obtained from the Health Research Ethics Committee at Stellenbosch University (N13/10/148) and permission from the Faculty of Medicine and Health Sciences at Stellenbosch University. No students refused consent to be included in the study.

## Results

In the MCQ examination, 50 students from the first semester (rotations one to four) were compared to 83 students in the second semester (rotations six to nine). The mean grade for the MCQ examination was significantly better (*p* = 0.03) in those with the PACK (72.3%, SD 8.6) compared to those without (68.8%, SD 9.6).

In the OSCE examination, 75 students in the first semester examination (rotations one to three and the sixth-year students that completed the rotation the previous year) were compared with 83 students in the second semester examination (rotations six to nine). The mean grade for the OSCE was significantly better (*p* < 0.001) in those with the PACK (64.4%, SD 7.0) compared to those without (58.8%, SD 10.9).

The qualitative exploration of how students experienced using the PACK is presented below as a series of themes.

### Understanding of the context

The PACK guide was not the only reference material that practitioners were exposed to in the clinical setting:

‘In the ARV [*anti-retroviral*] clinic we had the doctor, they would show us the [*HIV*] guidelines, with respect to ARV like follow up with patients, management and side effects, complication, staging, all of that.’ (Medical student, final phase, FGI1)

Some students felt that doctors should consult more disease-specific guidelines or the underlying evidence and not an integration of the evidence in a decision-support tool that was originally developed for nurses:

‘I’m purely speaking out of a doctor’s perspective, is that one would rather consult a different resource than a PACK guideline, primary health care guideline for my management of patients. If I would want to say ok, I’ve got a hypertensive, I would rather go and look up something like the newest hypertensive South African society.’ (Medical student, final phase, FGI2)

Students saw the benefits of the guide in terms of helping nurses who had less training as practitioners and in terms of defining the scope of practice for nurses and primary care doctors. The guide helped to define the continuum of care pathway from the nurse practitioner to the primary care doctor to the referral hospital:

‘I think they [*nurses*] would make the best use of these kind of books because they are running the clinic by themselves. They are sisters and when the doctor comes they’ll know who to refer to, but for us, I don’t know.’ (Medical student, final phase, FGI5)

### Views on the structure and content of the Practical Approach to Care Kit

Students were appreciative of the PACK tool as it helped them with an approach to common undifferentiated problems that they had not encountered in the tertiary hospital:

‘I really like the PACK guideline for things such as knowing what to do with a patient that’s unconscious, that has a cough or a cold, you know. The basic things that you don’t learn in internal medicine, because I mean, final year and you don’t know how to treat a cold, now I do.’ (Medical student, final phase, FGI3)

In addition, they appreciated that it defined what medication was actually available in the primary care setting and gave clear guidance on dosages, prompted them to think about the red flags that required urgent attention, used simple language, supported decision-making in a stepwise process and was not just a list of recommendations. The guide also encouraged a more thorough and comprehensive approach and could act as a checklist for everything that needed to be done. Students also liked the explanation of how the guide was linked to the underlying evidence. A few students complained that it was too big to carry around and that they struggled with the cross-referencing between sections. Students thought that the guide should be available in an app or e-version and not just paper-based.

### Orientation and training

Students agreed that orientation to the PACK was needed in the introduction to the module so that one felt more confident to use it in the clinical setting:

‘At least, right I know this section is diabetes, this is cardiovascular care. So when you do eventually use it in the clinic, since you already gone through it before, you really know where about you gonna go with it. It just makes using the PACK guideline a lot more user-friendly.’ (Medical student, final phase, FGI4)

Some felt that the workshops, facilitated by nurses, during the clinical rotation took them away from seeing real patients. There was some ambivalence about the facilitation from nurses as nurses were perceived to be less clinically knowledgeable and were unable to address questions that went beyond learning to just follow the tool. Students felt that some nurses were trying to teach them how to navigate the tool more than teaching them the content of clinical decision-making. Some nurses emailed answers to questions that they could not deal with in the workshop. This is in line with usual practice in the in-service PACK training programme where trainers are specifically encouraged to normalise that it’s not possible to know the answer to every clinical question, or be able to summarise everything in one guide, but that instead one needs to establish mechanisms and engage trusted sources to help address outstanding or particularly complex clinical issues.

Doctors, however, did not necessarily facilitate in a different way to nurses. Some students felt that the guide was self-explanatory and could be used for self or small group study without the need for a facilitator.

### Use in the consultation

The student consultation can be regarded as a triad involving the patient, the student and the supervising doctor. The PACK could be seen as a new element in the centre of this triad, which might be used in a variety of ways.

#### Student–supervisor interactions

The tool might be used in the dialogue between student and supervisor to discuss the consultation and develop a management plan. The tool then acted as an evidence-based benchmark for both students and practitioners. Some of the students appeared to use the tool as an additional authority in this discussion and juxtaposed the tool’s evidence-based recommendations against the practitioner’s usual practice. This could be a way of challenging the practitioner’s clinical practice as well as a way of developing the student’s clinical decision-making:

‘There was a discrepancy between what I wrote down because I used the PACK guideline and what he usually worked with. Say for example using prednisone in an acute gout attack. And then he actually said, ‘OK, so what does your PACK guideline say?’…So we had a look at the PACK guideline and then he saw OK well now, evidence based medicine says ‘Prednisone you give in an acute attack’. They don’t usually do that. So he just said we can leave it as such. He didn’t use it actively.’ (Medical student, final phase, FGI3)

#### Supervisor–patient interactions

It appeared that few nurses or doctors explicitly used the tool in their own consultations. This could be because they were already ‘unconsciously competent’ and had integrated the approach into their decision-making or could be because of a fear of appearing incompetent in the consultation in front of the patient by referring to a tool. Established practitioners appeared to have a set way of dealing with common problems that might differ from the tool’s recommendations. Students appeared to feel more comfortable to learn from the tool when its use was role modelled by their supervisor:

‘With regards to PACK, they didn’t use it, but … they know a lot. So they almost, not like that they don’t need PACK, but they’ve kind of been giving that same medication all the time.’ (Medical student, final phase, FGI6)

‘It’s actually quite good if you are the doctor that our site coordinator she would take it out of every single consultation just to check the contraindications or the interactions with other drugs. Every single consultation she would take it out. It’s was nice. You learn like that.’ (Medical student, final phase, FGI6)

#### Student–patient interactions

Some students used the tool during their consultations with patients, particularly when they had to share consulting rooms and collaborate on a consultation. The tool was then used as a basis for observation, feedback and peer learning:

‘The limited space, massive influx of patients and you really feel like you are in a warzone, but I mean, like there were 2 or 3 days … where we actually had to share consultation rooms and then we learn from each other. So he maybe … he corrected me and I corrected him.’ (Medical student, final phase, FGI4)

Some students struggled to refer to the tool in front of the patient because they felt this reinforced their lack of competence. Some excused themselves and then referred to it outside the room, while others managed to overcome this discomfort:

‘If I go to a doctor and he takes out a book, I’m gonna be like “well done, you actually looking up and making sure”, but some other people would be like “oh my goodness, this doctor doesn’t know what he’s doing”.’ (Medical student, final phase, FGI6)

‘The PACK helped a lot. In the beginning it did feel a bit like it takes a lot of the time during the consultation and you feel self-aware that you taking out a book in front of the patient but I didn’t feel like its … why must I ask the patient to excuse, like excuse the room, or I go out the whole time. It’s very unprofessional.’ (Medical student, final phase, FGI6)

However, when the guide was used in front of patients, they actually seemed to find this helpful and did not interpret it as a sign of incompetence:

‘We used it in front of the patient because it somehow gave the patient also that mindset that at least she’s double checking. She’s not just writing anything up. So I didn’t mind using it in front of the patient.’ (Medical student, final phase, FGI6)

Some student interns already felt that they were ‘consciously competent’ in managing patients and did not need to refer to a tool. They felt the tool would be more useful for nurses or more junior students:

‘I think that PACK is a good thing to have formulated and it will help a lot of health care workers working in primary care. I don’t think that it’s designed for us doctors or future doctors. The thing is we kind of spend 5 years learning all the conditions and we know how to diagnose hypertension, what to do if the patient [*has*] TB.’ (Medical student, final phase, FGI5)

Other students, however, did acknowledge that the guide was useful when they were ‘consciously incompetent’ and therefore unsure about what to do. Others found it useful to prompt them to make a more complete assessment and management plan:

‘You can’t go and check your guideline the whole time, after every patient. That’s a little impractical, but when you’re unsure about something.’ (Medical student, final phase, FGI5)

Interestingly, although students were present to learn and be trained, they were also pressurised by the workload and felt there was not enough time to consult a tool, particularly in the emergency centre:

‘So we had no real chance to use it in casualty and so we’re only actually experienced it during training sessions. Which is a pity … would be nice to use it a bit more but we … yea … didn’t get much of opportunity.’ (Medical student, final phase, FGI3)

Some students actually felt that using a guide would inhibit them from critical thinking and developing decisions based on their own internalised knowledge:

‘Something that we brought up with the nurses that helped us with going through the PACK guideline is that we’re afraid that this might hinder our thinking. You get what I’m saying? It’s there in the book, so I’m just going to page through it.’ (Medical student, final phase, FGI4)

Students recognised that it did not always anticipate the complexity of specific individuals and could not replace the need for critical thinking, judgement and experience.

### Reflection-on-action

Some students found it more useful to refer to the guide after the consultation was finished to check whether they had done the right thing or had been sufficiently comprehensive. Others used it to observe and make sense of decisions made by the doctor:

‘I used mine when I did my patient write-ups. I didn’t think doctors can’t make use of it at all. I think it will be more of a checking, like to check themselves than they actually … the patient’s gonna come in and they have hypertension and they have backache.’ (Medical student, final phase, FGI5)

A few students studied the PACK as if it were a textbook.

‘I don’t know how everyone else use the PACK … I sit down at a desk, go through the stuff and almost study it.’ (Medical student, final phase, FGI5)

## Discussion

Introduction of the PACK was associated with a significant improvement in student’s performance in primary care, which was particularly seen in the OSCE examination results. It is hoped that this would translate into better preparation of medical students for internship and community service to work alongside nurse clinicians in a task-sharing context.

Use of the PACK tool to support the development of competence can be interpreted in relation to the four stages of competence model (Table 1).^7^ Students who were ‘consciously incompetent’ used the PACK in the consultation to guide their approach to unfamiliar symptoms and conditions. These students appeared to have insight that they were still learning and in need of such a resource. Sometimes they used the tool covertly in the consultation as they feared that their incompetence would be made visible. Patients on the other hand appeared reassured by the explicit use of evidence in the consultation.

Students who were more ‘consciously competent’ would use the PACK to check on whether their approach had been correct after the consultation. These students recognised that their knowledge or approach might be incomplete and needed to be verified. This was particularly the case when they were expected to present a patient for assessment.

Some students believed they were already ‘unconsciously competent’ and did not think they needed the tool, although they could see its value for more junior students and nurses. Some of them appeared to believe that the use of a tool might actually impede the development of competence, because decisions would not be derived from one’s own knowledge base and critical thinking.

Students observed that most of their tutors did not openly use the PACK, which reinforced the concept that competence implies not having to use a tool. There were a few exceptions of experienced tutors that openly used the PACK to ensure they were up to date and to avoid errors. The predominant view amongst doctors, however, seemed to be that one should not explicitly use a tool and should rely on one’s unconscious competence, which was the product of experience in the context and prior training. From an educational viewpoint, therefore, it may be necessary to motivate the tutors to model explicit use of the tool in order to ensure that students feel comfortable to use it in the clinical setting. Use of the PACK in this way might enable tutors to deconstruct their ‘unconscious competence’ for students that were learning at a ‘consciously incompetent’ stage. In addition, use of PACK by the tutors could also model the need for all doctors to foster a part of their professional identity that is consciously incompetent and committed to lifelong learning. This is important to ensure that students learn how to make decisions and manage patients in primary care in a way that is aligned with the latest evidence and resources available.

Use of the PACK enhanced the impact of teaching by busy clinicians in the primary care environment. This may have been because it enabled students to engage with self-directed learning, self-assessment and evidence-based decision-making without being fully dependent on the tutor.^8^

Doctors are known to have an ambivalent relationship with guidelines in clinical practice.^14,15^ Guidelines may be seen as limiting individual autonomy, lacking application to the complexity of individual patients, implying a lack of competence or negating clinical practices that have been developed over time in a specific context. It was interesting to note that even medical students in their final year had already assimilated many of these attitudes. Guides were seen as necessary for those who were still learning to be competent or who had insufficient knowledge to solve clinical problems themselves, rather than applicable to all clinicians. This is reinforced by the fact that nurses refer patients to doctors when the patient’s problem is not covered by the guide. As PACK was initially developed to support nurses making clinical decisions in the primary care context, it could be that the format was less attuned to the higher level clinical reasoning expected of doctors.

It is also important in developing a transformative approach to learning^5^ to role model being open to change clinical practice in light of new evidence. Being a leader and a change agent implies an openness to innovate and improve. Unconscious competence is not a static state as new evidence may require us to cycle through earlier stages of competence again as we assimilate new knowledge into practice.

The quantitative study design was limited by excluding some of the rotations from the analysis in order to avoid contamination with the intervention. This reduced the sample size and power of the study, although significant differences were still seen. The results need to be interpreted in terms of improvement in performance that may be seen from the first to the second semester with increasing seniority. The study design did not allow for this to be adjusted for in the analysis. The improvement in OSCE mean score, however, was greater than what had been observed in previous years and what might be expected from seniority alone.

This study supports the use of the PACK at an undergraduate level and suggests that it may be more valuable to introduce it earlier when students are first exposed to clinical primary care and not only in the final phase. The PACK is now available electronically, although initial familiarisation with the structure and content of the tool may be better using the paper version.

The supervisors should be trained as educators to model use of the PACK in their own clinical practice and in their educational conversations with students in order to deconstruct their clinical decision-making and model being change agents.

## Conclusion

The performance of undergraduate students in primary care improved following the introduction of the PACK in their final phase using an inter-professional model of training. The PACK should be used going forward, but introduced in the early phase. Supervisors should be trained further in how to incorporate use of the PACK in their practice and educational conversations.
